# Isolation and Molecular Characterization of *Brucella* Isolates in Cattle Milk in Uganda

**DOI:** 10.1155/2015/720413

**Published:** 2015-02-22

**Authors:** Denis Rwabiita Mugizi, Shaman Muradrasoli, Sofia Boqvist, Joseph Erume, George William Nasinyama, Charles Waiswa, Gerald Mboowa, Markus Klint, Ulf Magnusson

**Affiliations:** ^1^College of Veterinary Medicine, Animal Resources and Bio-Security, Makerere University, P.O. Box 7062, Kampala, Uganda; ^2^Division of Bacteriology and Food Safety, Department of Biomedical Sciences and Veterinary Public Health, Faculty of Veterinary Medicine and Animal Science, Swedish University of Agricultural Sciences, P.O. Box 7028, 75007 Uppsala, Sweden; ^3^College of Health Sciences, Makerere University, P.O. Box 7062, Kampala, Uganda; ^4^Section of Clinical Bacteriology, Department of Medical Sciences, Uppsala University, 75185 Uppsala, Sweden; ^5^Division of Reproduction, Department of Clinical Sciences, Faculty of Veterinary, Medicine and Animal Science, Swedish University of Agricultural Sciences, P.O. Box 7054, 75007 Uppsala, Sweden

## Abstract

Brucellosis is endemic in livestock and humans in Uganda and its transmission involves a multitude of risk factors like consumption of milk from infected cattle. To shed new light on the epidemiology of brucellosis in Uganda the present study used phenotypic and molecular approaches to delineate the *Brucella* species, biovars, and genotypes shed in cattle milk. *Brucella abortus* without a biovar designation was isolated from eleven out of 207 milk samples from cattle in Uganda. These isolates had a genomic monomorphism at 16 variable number tandem repeat (VNTR) loci and showed in turn high levels of genetic variation when compared with other African strains or other *B. abortus* biovars from other parts of the world. This study further highlights the usefulness of MLVA as an epidemiological tool for investigation of *Brucella* infections.

## 1. Introduction

The genus* Brucella* has ten recognized species with more than 90% DNA homology [1, 2]. These species cause brucellosis that is of economic and public health importance in terrestrial and aquatic animals and humans [1, 3]. Species of* B. melitensis*,* B. abortus*, some* B. suis* biovars,* B. canis*,* B. ceti*, and* B. inopinata* are zoonotic and in humans the infection causes a debilitating disease with relapsing fever and flu-like symptoms with multiple organ involvement [4–6]. In cattle* Brucella* causes abortions, placentitis, orchitis, mastitis, and prenatal death [5, 7].

Brucellosis in cattle is almost exclusively caused by* B. abortus* [8], but* B. melitensis* and* B. suis* have been implicated in some herds [9, 10] making the vaccination of cattle using vaccines targeting only* B. abortus* less effective in preventing brucellosis in cattle and transmission to humans [11, 12].* Brucella* biovars and genotypes are known to be regionally restricted in their distribution [13] but the evolution of international travel and trade and changing ecosystems have led to introduction of new biovars and genotypes into regions and hosts where they were not previously found [3]. A study done in Uganda in 1958 isolated* B. abortus* biovar 3 from a human patient [14] and in the neighboring Kenya* B. melitensis* biovar 1 and* B. abortus* biovar 3 have been isolated from cattle [9]. Phylogenetic analysis of* B. melitensis* isolates in Kenya showed a high degree of homology with isolates in Israel and the* B. abortus* isolates closely resembled that isolated in Uganda. In a related study in Egypt,* B. abortus*,* B. suis*, and* B. melitensis* were isolated from cattle and all had a high level of phylogenetic variability within each species although the isolates used in these studies were few [15]. The above findings indicate a complex epidemiology of brucellosis in cattle in the region and call for refined diagnostic methods beyond phenotypic typing.

High resolution phenotypic and molecular approaches have been developed for* Brucella* speciation, biotyping, and epidemiological trace-back [16, 17]. To date, advanced molecular technologies have not been widely used in low income countries where brucellosis is endemic in livestock and humans [7, 18]. Thus, information on the prevailing* Brucella* species, biovars, and genotypes/strains in such areas of endemicity may shed new light on the epidemiology of* Brucella *infection and the species and biovars circulating. Besides this generic scientific rationale for undertaking such investigations, increased understanding of the* Brucella *epidemiology is critical for refining control of brucellosis in resource weak countries where the same measures as in high income countries cannot be applied.

In northern and eastern Uganda where this study was performed, there was a considerable mixing of livestock species during years of insurgency in the 1990s, presenting ideal conditions for inter- and intraherd transmission of diseases such as brucellosis. Indeed, high herd and individual animal seroprevalences of up to 27% and 7.5%, respectively, were recorded in a recent survey in the region [19]. This high prevalence may pose a severe threat to public health as previous studies around the capital of Uganda suggest milk or milk products from cows as a major source of* Brucella* infection in humans [20, 21]. The present study aimed at isolating and molecular-typing* Brucella* from cattle milk in northern and eastern Uganda for better understanding of its epidemiology.

## 2. Materials and Methods

### 2.1. Study Design and Collection of Samples

Milk samples were collected from 207 lactating cows in urban and periurban areas of Gulu and Soroti towns of northern and eastern Uganda from May 2011 to March 2012 for isolation of* Brucella*. A total of 110 individual cow milk samples were collected from 72 herds in Gulu and a total of 97 individual cow milk samples were collected from 33 herds in Soroti. These herds were part of the 166 herds whose animals had been screened for brucellosis and were within a radius of 15 Km in both Gulu and Soroti towns, with the two towns being 200 Km apart. The cattle from which milk samples were taken had been screened for* Brucella* antibodies and in total 17 of the 207 cows were seropositive. In both towns, the seropositive herds from which the milk samples were taken were near each other. The numbers and selection of households and animals included are described in detail previously [19]. Midstream milk samples were collected from all quarters with 10–20 mL collected from each teat into sterile 100 mL falcon tubes. The samples were transported chilled to Makerere University College of Health Sciences microbiology laboratory, Kampala, Uganda, kept at 4°C, and cultured within three days.

### 2.2. Sample Preparation,* Brucella* Culturing, and Biotyping

The milk was centrifuged at 3000 ×g at 4°C for 15 minutes and the pellet and supernatant were plated on both Farrel and Centro de Investigación y Tecnología Agroalimentaria (CITA) selective media supplemented with calf serum [22]. Briefly, Farrel's medium was prepared from* Brucella* medium base (Oxoid, UK), sterilized at 121°C for 15 minutes, and supplemented with* Brucella* selective supplement (Oxoid, UK) according to the manufacturer's instructions. The CITA medium was prepared according to De Miguel et al. [22]. Inoculated plates were incubated at 37°C for 8 days in a 5–10% carbon-dioxide incubator and read every 24 hours from day three of incubation for colony growth. Resultant colonies were subcultured and biotyped based on their colony morphology, serum and carbon-dioxide requirement for growth, hydrogen sulphide production, urease activity, oxidase test, and growth in presence of dye basic fuchsin and agglutination of anti-*Brucella* IgG monospecific sera A (Animal Health and Veterinary Laboratories Agencies, Weybridge, UK), according to the OIE Terrestrial Manual [8]. Representative colonies are stored at −80°C in 20% glycerol for long-term storage.

### 2.3. Genomic DNA Extraction and Real-Time PCR Detection

The colonies that conformed to all the above phenotypic characteristics of* Brucella* were subjected to genomic DNA extraction using the Norgen bacterial genomic DNA isolation kit (Norgen Biotek Corp., Ontario, Canada). The extracted* Brucella* genomic DNA was used to run a real-time multiplex PCR assay with oligonucleotide primers, probes, reaction mixture, and PCR conditions according to Probert et al. [23]. Amplification and real-time fluorescence detection were done on the Rotor-Gene 3000 real-time PCR machine (Corbett Research-Corbett Life Sciences, Mort Lake Australia). Three positive and two negative controls were included in each run. When the cycle threshold (CT) value of the samples was ≤40, samples were evaluated as positive. This real-time multiplex PCR was designed to detect* Brucella* at both the genus and species levels for* B. abortus* and* B. melitensis* since it has the genus specific probe and the species specific probes for the two* Brucella* species commonly infecting cattle.

### 2.4. *Brucella* Species Confirmation

Positive samples on real-time PCR were analysed further for* Brucella* speciation using the Bruce-ladder multiplex PCR assay kit (Ingenasa, Spain). The oligonucleotide primers, reaction mixture, and PCR conditions were performed according to the manufacture's conditions in conformity with López-Goñi et al. [16]. However amplification was done in a MyCycler thermal cycler (BioRad).

### 2.5. Characterization of Isolates by MLVA Genotyping

**Figure 1:**
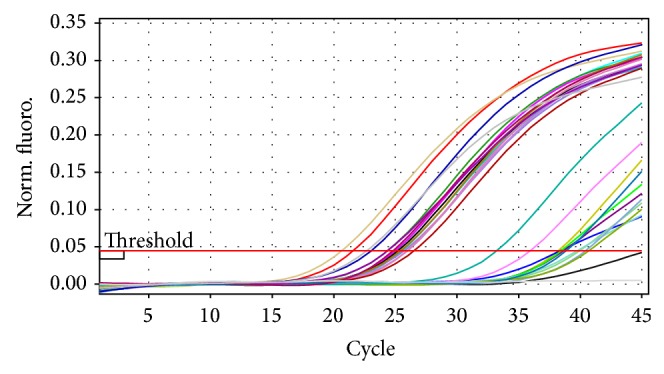
Triplex real-time PCR amplification pattern using the* Brucella* genus probe. Fluorescence ratio is plotted against the number of PCR cycles to monitor amplification in real-time mode. Isolates with weak Ct values (29.18 and above) had* Brucella*-like phenotypic characteristics and were included in this assay.

Genotyping was performed using the Multiple Locus Variable Number Tandem Repeat Analysis (MLVA), using the 16-primer-pair assay [17, 24–26]. The oligonucleotide primer pairs incorporated in the* Brucella* MLVA 16 assay target both the conserved and highly discriminatory regions of the* Brucella* genome. Each sample was run on three prescribed MLVA panels. Panel 1 consisted of moderately polymorphic minisatellite primer pairs targeting the highly conserved genomic regions of different* Brucella* species (bruce 06, bruce 08, bruce 11, bruce 12, bruce 42, bruce 43, bruce 45, and bruce 55). Panel 2A (bruce 18, bruce 19, and bruce 21) and panel 2B (bruce 04, bruce 07, bruce 09, bruce 16, and bruce 30) consisted of microsatellite primer pairs targeting the discriminatory genomic regions. The PCR amplification and genotyping were done according to le Flèche et al. [17] with only a modification in the total reaction volume to 30 *μ*L. At each run,* B. suis* reference strain REF. 1330 (from Bruce-ladder kit) was included as shown in Figure 1.

### 2.6. Gel-Electrophoresis Analysis of Panel 1 and Panel 2 Loci Amplification Products

Five microliters of the panel 1 and panel 2 loci amplification products were loaded into 3% and 2% agarose gel containing ethidium bromide (0.5 *μ*g/mL), respectively, to visualize the banding pattern in the samples and positive controls, under UV illumination. The agarose gel was run on 8 V/cm current, and a 100 base pair and 20 base pair ladders (BioRad) were included per run for panel 1 and panel 2, respectively.

### 2.7. Sequencing the VNTR Locus Amplicons

In order to identify repeat copy number variation among the isolates in question the resulting PCR products were sequenced for each VNTR locus at Macrogen, Netherlands. Sequences were viewed using BioEDIT version 7.0.9.0. Sequencing was performed in both directions using the M13-primers according to Applied Biosystems (ABI). Since each of the VNTR locus primers was tagged with M13 primer ([M13-Forward] 5′-GTAAAACGACGGCCAGT-3′ and [M13-Rev] 5′-GCGGATAACAATTTCACACAGG-3′), this increases each VNTR locus PCR product size by 39 base pairs.

### 2.8. Analysis of MLVA Sequence Data

The MLVA PCR products were sequenced per loci and the forward and reverse sequences were assembled into a contig in the Bionumerics software version 5.0 (Applied Maths, Belgium). The M13 primer tags were trimmed from the contig and the allele designation was determined by comparing the fragment size with the published allele numbering system (version 3.6 http://mlva.u-psud.fr *Brucella* support website for MLVA typing). The number of tandem repeats per loci was queried in the* Brucella* MLVA 2012 public database (http://mlva.u-psud.fr/mlvav4/genotyping/) accessed on February 21, 2014, for genotyping of our isolates. The closest related known strains were determined based on the genetic distance (the minimum number of changes in the number of repeats of any locus that converts one genotype to another).

## 3. Results

### 3.1. Biotyping

Based on the biotyping (Table 1),* B. abortus* biovar 1, 3, or 7 was isolated in 11 (5.3%) out of 207 milk samples. These 11 positive samples were all from seropositive cows (i.e., 11 of 17). The colonies being smooth eliminated* B. canis* and* B. ovis* which have rough colonies. Production of hydrogen sulfide eliminated* B. melitensis*,* B. ceti*,* B. microti* and* B. abortus* biovars 5 and 6, and* B. suis* except* B. suis* biovar 1. Ability to grow in absence of serum eliminated* B. abortus* biovar 2 that generally requires serum for growth.* Brucella abortus* biovar 2,* B. neotomae*, and* B. suis* biovar 1 were eliminated by their inability to grow in basic fuchsin. Agglutination with anti-*Brucella* monospecific sera A eliminated* B. abortus* biovars 4 and 9.

### 3.2. Molecular Characterization

#### 3.2.1. *Brucella* DNA Detection by Real-Time PCR

**Figure 2:**
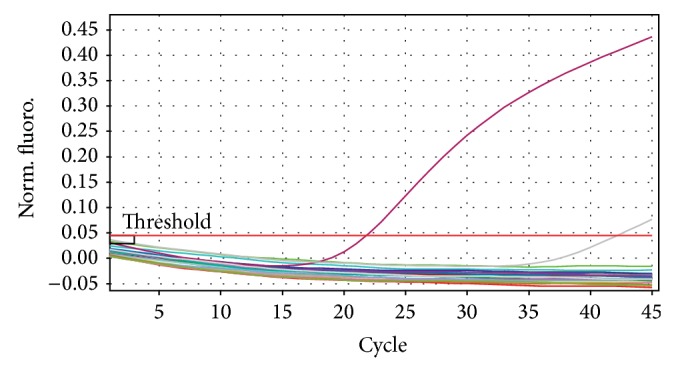
Triplex real-time PCR amplification pattern using the* B. melitensis* probe. Fluorescence ratio is plotted against the number of PCR cycles to monitor amplification in real-time mode. Only* B. melitensis* (positive control) was picked by this probe.

**Figure 3:**
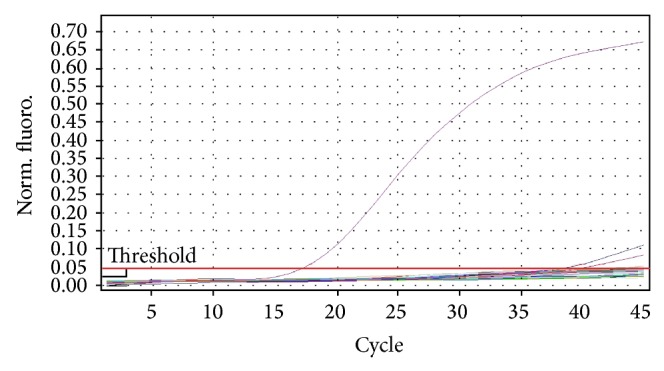
Triplex real-time PCR amplification pattern using the* B. abortus* probe. Fluorescence ratio is plotted against the number of PCR cycles to monitor amplification in real-time mode. Only* B. abortus* (positive control) was picked with a strong Ct value by this probe.

DNA from all the 11 isolates that was judged as* B. abortus* by the biotyping was detected as* Brucella* DNA by the* Brucella* genus probe in the triplex real-time PCR (Figure 1). However the triplex real-time PCR was unable to detect the* Brucella* species involved using its* B. melitensis* probe (Figure 2) and* B. abortus* probe (Figure 3).

#### 3.2.2. *Brucella* Species Confirmation by Bruce-Ladder

**Figure 4:**
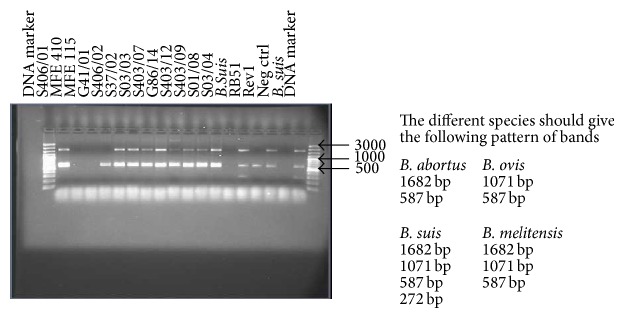
Bruce-ladder multiplex PCR agarose gel picture used to confirm the* Brucella *species isolated. Extreme left and right lanes are for 100 bp molecular weight marker; from left to right: lanes 2, 5–13 are for DNA from isolates detected as* Brucella* with strong Ct values; lanes 3, 4, and 14 are for DNA from* Brucella*-like isolates with weak Ct values; lanes 15 and 19 are for* B. suis* positive control DNA; lane 16 is for RB 51 positive control DNA; lane 17 is for Rev 1 positive control DNA; and lane 18 is for PCR grade water (negative control).

DNA from all the 11 isolates that were confirmed as belonging to the genus* Brucella* in the triplex real-time PCR was detected as* B. abortus* DNA by the Bruce-ladder multiplex PCR (Figure 4).* B. abortus* gives two bands of 1682 bp and 587 bp on Bruce-ladder PCR.

#### 3.2.3. MLVA Genotyping

**Figure 5:**
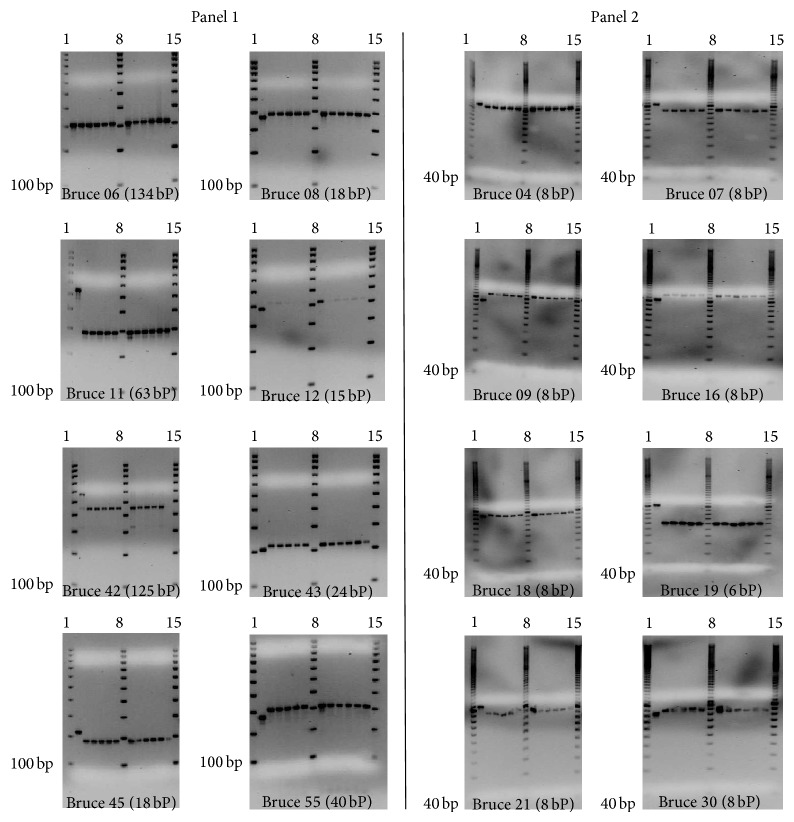
MLVA amplification pattern of isolates in this study and a* B. suis *as control. Lanes 1, 8, and 15 show DNA marker, lane 2 in each gel shows pattern for* B. suis*, and lanes 3–7 and 9–14 show the amplification pattern of isolates 1–11 (G41/01, S406/02, S37/02, S03/03, S403/07, S406/01, G86/14, S403/12, S403/9, S09/08, and S02/10).

The MLVA-16 assay revealed that none of the 11 isolates did match any of the* Brucella* isolates in the* Brucella* MLVA 2012 database but closely resembled the former* B. abortus* biovar 7 strain 07-994-2411 from Kenya (Table 2). All isolates obtained were monomorphic at all loci as shown in Table 2 and Figure 5. We designated these isolates as UG* Ba-m* because they were isolated from cattle in Uganda and had a* B. abortus* profile on most biotyping assays but resembled both* B. melitensis* and* B. abortus* at genotyping. Both of the UG* Ba-m* isolates and the* B. abortus* strain 07-994-2411 showed close resemblance to a human* B. melitensis* biovar 1 (strain BCCN87-92) strain isolated from USA. When compared on MLVA-8 panel 1 loci the genetic distance was only zero between UG* Ba-m* isolates and* B. abortus* strain 07-994-2411 and one was between UG* Ba-m* isolates and* B. melitensis* biovar 1 strain BCCN87-92.

## 4. Discussion

Here we present for the first time phenotypic and molecular characterization of* Brucella* isolates from cattle milk in Uganda. These results contribute to better understanding of geographical transmission patterns of* Brucella* in cattle in Uganda and are important if specific control measures are to be implemented in the future. In Uganda, most of the milk is marketed unprocessed through the informal milk marketing linkages, thus acting as a potential source of human brucellosis infections.

All UG* Ba-m* isolates were from* Brucella* seropositive cattle conforming to the well-known fact that* Brucella* infected lactating female animals shed the bacteria in their milk since the organism relocates to the udder from the pregnant uterus upon delivery [27]. This has public health implications in the region since most of the milk is consumed unpasteurized. One third of the seropositive cattle were not shedding the* Brucella* in the milk suggesting that these animals either had cleared the infection or were chronically sick, thus not shedding the bacteria as shown in a study by Capparelli et al. [28]. All UG* Ba-m* isolates being from seropositive cattle suggest an active infection. Notably,* Brucella* was not isolated from milk from any of the seronegative cows. This suggests that seroconversion precedes shedding of the* Brucella* in milk, and thus serological tests can be sufficient in predicting possible shedders.

The phenotypic characteristics of all UG* Ba-m* isolates matched those of* B. abortus* biovars 1, 3 or the former biovar 7. All isolates being* B. abortus* conform to the well-known fact that* B. abortus* is the predominant species in cattle [14]. Furthermore, all 11 UG* Ba-m* isolates having the same phenotypic profile suggest that they belong to the same biovar attesting to the suggestion of regional predominance of certain* Brucella* biovars in Africa, for instance, predominance of* B. abortus* biovar 6 in nomadic cattle in Western Sudan [29],* B. abortus* biovar 3 and* B. melitensis* biovar 1 in cattle in Kenya [9], and* B. melitensis* biovar 3 in ruminants in Egypt [15]. However the numbers of isolates in these studies were too few to make a solid basis for generalization.

Detection of all the 11 UG* Ba-m* isolates as* Brucella* by the* Brucella* genus specific probe in the triplex real-time PCR with strong signals confirmed them as* Brucella*. The inability of the* B. melitensis *probe in the triplex real-time PCR to detect the isolates as* B. melitensis* suggested that they are not* B. melitensis. *The inability of the* B. abortus* species specific probe in the triplex real-time PCR to detect the isolates as* B. abortus* suggested that they are not* B. abortus* biovars 1, 2, 3, 4, 5, 6, and 9 but could be the former* B. abortus *biovar 7 since the primers used were not targeting* B. abortus* biovar 7 [23, 30].

All the 11 UG* Ba-m* isolates were confirmed as* B. abortus* by the Bruce-ladder multiplex PCR. The inability of the triplex real-time PCR to detect these isolates at its* B. abortus* species specific probe contrary to the Bruce-ladder multiplex PCR suggests that these isolates belong to the former* Brucella abortus* biovar 7. This suggestion is based on the fact that the triplex real-time PCR was not designed to detect the former* Brucella abortus* biovar 7 with its* B. abortus* species specific probe (detecting only* B. abortus* biovars 1, 2, 3, 4, 5, 6, and 9), and the Bruce-ladder multiplex PCR was designed to detect all the* B. abortus *biovars including the former* B. abortus* biovar 7 [16, 23, 30]. This suggests that a diphasic PCR protocol involving the triplex real-time PCR by Probert et al. [23] and the Bruce-ladder multiplex PCR by López-Goñi et al. [16] could be used to replace the risky procedure of identifying the former* B. abortus* biovar 7 using the conventional biotyping methods by detecting particularly its agglutination with monospecific anti-sera A and M and its growth in both basic fuchsin and thionin dyes. This protocol needs, however, to be tested on all the former* B. abortus* biovar 7 isolates.

The evidence adduced using a combination of phenotypic and molecular approaches designated all UG* Ba-m* isolates as atypical* B. abortus* without a biovar designation. All the isolates were monomorphic at molecular analysis, which could be due to the isolates being from a small geographical region of approximately 15 Km radius per region (data not shown here) and having been collected in a short time frame making it possible for all the isolates to be from a common source as animals mix in the grazing grounds. The genetic monomorphism observed is partly congruent with that observed in five* B. melitensis* biovar 1 isolates obtained from bovine milk in central Kenya by Muendo et al. [9], although their finding was in a different* Brucella* species. Our results are further supported by findings by Garin-Bastuji et al. [31] who found similar monomorphism in isolates in Mongolia isolated 5 years apart in the same region. The genetic monomorphism exhibited at the minisatellite and microsatellite loci that are otherwise polymorphic even in highly genetic homogenous species like* Brucella* suggests that there is one or very few circulating strains of* Brucella* in this region of Uganda attesting to the regional predominance of* Brucella* biovars and strains.

The closest known strain for the 11 UG* Ba-m* isolates was a* B. abortus* strain 07-994-2411 isolated from cattle in the neighboring Kenya in 1963. This strain has no biovar designation but was formerly known as* B. abortus* biovar 7, before biovar 7 was suspended from the* Brucella* nomenclature (International Committee on Systematic Bacteriology, 1988).* B. abortus* biovar 7 was suspended from* Brucella* nomenclature because the reference strain (63/75) was thought to be a mixture of* B. abortus* biovars 3 and 5. A recent study by Garin-Bastuji et al. [31] proposed the reintroduction of* B. abortus* biovar 7 in the approved list of bacterial names having identified* B. abortus* strains from Turkey, Mongolia, and Kenya that perfectly matched the former* B. abortus *biovar 7 characteristics.* B. abortus *biovar 7 can be differentiated from other* B. abortus* biovars by its ability to agglutinate with anti-A and anti-B monospecific sera.* B. abortus* biovar 7 has smooth colonies, does not require carbon dioxide for growth, produces hydrogen sulphide, is oxidase and urease positive, does not agglutinate with monospecific anti-sera R, grows in the presence of dyes thionin and basic fuchsin at a concentration of 20 *μ*g/mL, and is lysed by phages Tbilisi (Tb), Weybridge (Wb), Izatnagar 1 (LZ_1_), and R/C.

A genetic difference in the UG* Ba-m* isolates at 5 loci out of 16 polymorphic loci examined compared to the known closest related strain (07-994-2411) from Kenya in a period of half a century could be a result of mutations and proves the ability of MLVA to differentiate strains from different localities, a finding congruent with that of Verger et al. [2]. The observed genetic similarity between the Kenyan strain and the 11 UG* Ba-m* isolates compared to other isolates from distant places could be due to the cross-border transmission of* Brucella* in cattle that could have been facilitated by cattle rustling across the pastoral Karamoja subregion of Uganda and Kenya over the past years.

## 5. Conclusions

In conclusion, our findings suggest* B. abortus* without biovar designation (atypical* B. abortus*) as a cause of brucellosis in cattle in northern and eastern Uganda. The Ugandan isolates exhibited a single MLVA-16 pattern and show in turn high levels of genetic variation when compared with other African strains, highlighting the usefulness of MLVA as an epidemiological tool for investigation of* Brucella* infections. Furthermore, the ability of a diphasic PCR protocol involving the triplex real-time PCR by Probert et al. [23] and the Bruce-ladder multiplex PCR by López-Goñi et al. [16] to detect* B. abortus* could be used to replace the procedure of identifying the former* B. abortus* biovar 7 using the conventional biotyping methods.

## Figures and Tables

**Table 1:** Phenotypic characteristics of *Brucella* spp. isolated from cattle milk from northern and eastern Uganda in 2011-2012.

Sample ID	Serological result	Colony morphology	Serum requirement	CO_2_ requirement	Oxidase test	Urease activity	Agglutination with monospecific sera A	H_2_S production	Growth in basic fuchsin	Biovar
S406/01	+	S	−	−	+	+	+	+	+	1 or 3
S403/07	+	S	−	−	+	+	+	+	+	1 or 3
S03/03	+	S	−	−	+	+	+	+	+	1 or 3
S37/02	+	S	−	−	+	+	+	+	+	1 or 3
S406/02	+	S	−	−	+	+	+	+	+	1 or 3
G41/01	+	S	−	−	+	+	+	+	+	1 or 3
G86/14	+	S	−	−	+	+	+	+	+	1 or 3
S403/12	+	S	−	−	+	+	+	+	+	1 or 3
S403/09	+	S	−	−	+	+	+	+	+	1 or 3
S01/08	+	S	−	−	+	+	+	+	+	1 or 3
S02/10	+	S	−	−	+	+	+	+	+	1 or 3

S: smooth colonies.

**Table 2:** *Brucella* spp. isolates recovered from cattle milk samples in northern and eastern Uganda (2011-2012) and their MLVA 16 profile compared to that of some known *Brucella* strains from *Brucella *MLVA database and strains conforming to *B. abortus* former biovar 7.

Sample ID/strains	Year	Region	Host	Specimen	Number of repeats for the polymorphic panel 1 and panel 2 Bruce loci																Species	Biovar	Genotype
					Panel 1								Panel 2A			Panel 2B				
					6	8	11	12	42	43	45	55	18	19	21	4	7	9	16	30
G41/01	2011	Gulu	Cattle	Milk	2	4	2	12	3	2	3	3	5	9	6	4	2	9	8	4	*B*. *abortus *	ND	UG *Ba-m *
S406/02	2012	Soroti	Cattle	Milk	2	4	2	12	3	2	3	3	5	9	6	4	2	9	8	4	*B*. *abortus *	ND	UG *Ba-m *
S37/02	2012	Soroti	Cattle	Milk	2	4	2	12	3	2	3	3	5	9	6	4	2	9	8	4	*B*. *abortus *	ND	UG *Ba-m *
S03/03	2012	Soroti	Cattle	Milk	2	4	2	12	3	2	3	3	5	9	6	4	2	9	8	4	*B*. *abortus *	ND	UG *Ba-m *
S403/07	2012	Soroti	Cattle	Milk	2	4	2	12	3	2	3	3	5	9	6	4	2	9	8	4	*B*. *abortus *	ND	UG *Ba-m *
S406/01	2012	Soroti	Cattle	Milk	2	4	2	12	3	2	3	3	5	9	6	4	2	9	8	4	*B*. *abortus *	ND	UG *Ba-m *
G86/14	2011	Gulu	Cattle	Milk	2	4	2	12	3	2	3	3	5	9	6	4	2	9	8	4	*B*. *abortus *	ND	UG *Ba-m *
S403/12	2012	Soroti	Cattle	Milk	2	4	2	12	3	2	3	3	5	9	6	4	2	9	8	4	*B*. *abortus *	ND	UG *Ba-m *
S403/09	2012	Soroti	Cattle	Milk	2	4	2	12	3	2	3	3	5	9	6	4	2	9	8	4	*B*. *abortus *	ND	UG *Ba-m *
S09/08	2012	Soroti	Cattle	Milk	2	4	2	12	3	2	3	3	5	9	6	4	2	9	8	4	*B*. *abortus *	ND	UG *Ba-m *
S02/10	2012	Soroti	Cattle	Milk	2	4	2	12	3	2	3	3	5	9	6	4	2	9	8	4	*B*. *abortus *	ND	UG *Ba-m *
^*^REF 1330	—	USA	Swine	—	2	3	6	10	4	1	5	2	4	38	9	6	6	5	5	3	*B*. *suis *	Biovar 1	33 (MLVA-11)
^ a^BCCN#87-92	1997	USA	Human	—	2	4	2	12	4	2	3	3	9	36	7	2	4	3	3	5	*B*. *melitensis *	Biovar 1	126 (MLVA-11)
^ a^REF 544	1942	England	Bovine	—	4	5	4	12	2	2	3	3	5	42	8	3	5	3	4	5	*B*. *abortus *	Biovar 1	83 (MLVA-11)
^ a^REF 86/8/59	1959	England	Bovine	—	4	5	4	12	2	1	3	3	6	42	8	3	4	3	3	5	*B*. *abortus *	Biovar 2	80 (MLVA-11)
^ a^REF Tulya	1958	Uganda	Human	—	3	5	4	11	2	2	3	3	8	40	8	6	5	3	11	5	*B*. *abortus *	Biovar 3	64 (MLVA-11)
^ a^REF 292	1961	England	Bovine	—	4	5	4	12	2	2	3	2	6	42	8	3	4	3	3	5	*B*. *abortus *	Biovar 4	78 (MLVA-11)
^ a^REF B3196	1959	England	Bovine	—	3	5	3	12	2	2	2	3	7	42	8	6	7	3	3	3	*B*. *abortus *	Biovar 5	67 (MLVA-11)
^ a^REF 870	1959	Africa	Bovine	—	3	5	3	12	2	2	3	3	7	42	8	3	6	3	3	3	*B*. *abortus *	Biovar 6	66 (MLVA-11)
^ a^REF C68	1958	England	Bovine	—	3	5	3	12	2	2	2	3	7	42	8	6	6	3	3	3	*B*. *abortus *	Biovar 9	67 (MLVA-11)
^ a^BCCN#V1 (S19)	1943	USA	Bovine	—	4	5	4	12	2	2	3	3	6	42	8	3	5	3	3	5	*B*. *abortus *	Biovar 1	82 (MLVA-11)
^ a^BCCN#V5 (RB51)	—	USA	Bovine	—	4	5	4	12	2	3	3	3	6	42	8	3	7	3	3	5	*B*. *abortus *	Biovar 1	79 (MLVA-11)
^ a^13	2006-2007	Egypt	Cattle	Spleen	4	5	4	12	2	3	3	3	6	—	8	3	7	3	3	5	*B*. *abortus *	—	—
^ a^4	2002–2007	Egypt	Cattle	—	4	5	4	12	2	3	3	3	6	—	8	3	9	3	3	5	*B*. *abortus *	—	—
^ a^6-KEBa 1	2009	Kenya	Cattle	Abortion material	3	5	4	11	2	2	3	3	7	40	8	6	6	3	11	6	*B*. *abortus *	Biovar 3	34 (MLVA-8)
^ a^11-KEBa 2	2009	Kenya	Cattle	Vaginal discharge	3	5	4	11	2	2	3	3	7	40	8	6	5	3	12	5	*B*. *abortus *	Biovar 3	34 (MLVA-8)
^ a^BCCN#93-26	1993	Sudan	Dromedary	—	3	5	4	11	2	2	3	3	6	40	8	6	8	3	7	7	*B*. *abortus *	Biovar 3	63 (MLVA-11)
^ b^07-994-2411	1963	Kenya	Bovine	—	2	4	2	12	3	2	3	3	5	22	6	5	2	6	7	5	*B*. *abortus *	ND	ND
^ b^03-4923-239	2003	Turkey	Bovine	—	4	5	3	12	2	2	3	1	6	21	8	6	7	6	3	3	*B*. *abortus *	ND	ND
^ b^99-9971-135	1988	Mongolia	Bovine	—	4	5	5	12	2	2	2	2	6	21	8	5	6	4	3	3	*B*. *abortus *	ND	ND
^ b^99-9971-159	1993	Mongolia	Bovine	—	4	5	5	12	2	2	2	2	6	21	8	5	6	4	3	3	*B*. *abortus *	ND	ND

^a^Strains downloaded from *Brucella* MLVA database February 2014; http://mlva.u-psud.fr;  ^*^the reference strain used (from Bruce-ladder kit); ND: not designated; ^b^strains conforming to *B. abortus* former biovar 7.
